# Decision-Making under Risk of Loss in Children

**DOI:** 10.1371/journal.pone.0052316

**Published:** 2013-01-09

**Authors:** Sophie Steelandt, Marie-Hélène Broihanne, Amélie Romain, Bernard Thierry, Valérie Dufour

**Affiliations:** 1 Centre National de la Recherche Scientifique, Département Ecologie, Physiologie et Ethologie, Strasbourg, France; 2 Université de Strasbourg, Institut Pluridisciplinaire Hubert Curien, Strasbourg, France; 3 Laboratoire de Recherche en Gestion et Économie, EM Strasbourg Business School, Université de Strasbourg, Strasbourg, France; 4 SPRG, Centre for Social Learning and Cognitive Evolution, Scottish Primate Research Group, School of Psychology, University of St-Andrews, St. Andrews, United Kingdom; University of Granada, Spain

## Abstract

In human adults, judgment errors are known to often lead to irrational decision-making in risky contexts. While these errors can affect the accuracy of profit evaluation, they may have once enhanced survival in dangerous contexts following a “better be safe than sorry” rule of thumb. Such a rule can be critical for children, and it could develop early on. Here, we investigated the rationality of choices and the possible occurrence of judgment errors in children aged 3 to 9 years when exposed to a risky trade. Children were allocated with a piece of cookie that they could either keep or risk in exchange of the content of one cup among 6, visible in front of them. In the cups, cookies could be of larger, equal or smaller sizes than the initial allocation. Chances of losing or winning were manipulated by presenting different combinations of cookie sizes in the cups (for example 3 large, 2 equal and 1 small cookie). We investigated the rationality of children's response using the theoretical models of Expected Utility Theory (EUT) and Cumulative Prospect Theory. Children aged 3 to 4 years old were unable to discriminate the profitability of exchanging in the different combinations. From 5 years, children were better at maximizing their benefit in each combination, their decisions were negatively induced by the probability of losing, and they exhibited a framing effect, a judgment error found in adults. Confronting data to the EUT indicated that children aged over 5 were risk-seekers but also revealed inconsistencies in their choices. According to a complementary model, the Cumulative Prospect Theory (CPT), they exhibited loss aversion, a pattern also found in adults. These findings confirm that adult-like judgment errors occur in children, which suggests that they possess a survival value.

## Introduction

Individuals are regularly faced with fluctuations of their economic or general environment which are akin to situations of risk. When confronted to such random outcomes – the potential loss of money for example – they have to evaluate the odds of success, and to take the best decision. Natural selection likely shaped decision rules that help coping with risks [1], and it is expected that decision-making tends toward optimality [2], [3]. Decision-making under risk is a well-studied question in economics, where losses can be at stake. According to Knight [4] a risky situation is, as opposed to an uncertain one, a situation where probabilities are well known, like in a lottery in which the probability of occurrence of each outcome is known – for example: 50% chances to win. A risky context is also different from an ambiguous one where only partial information is available about the odds – for example when the only subject's knowledge about the lottery outcomes is a chance of winning comprised between 50% and 100% [5].

In the realm of decision making under risk, the classical models in economics have long been based on the assumption that humans are rational decision-makers: when faced with a lottery game individuals should evaluate the odds of winning and losing before buying a ticket, and they should only chose the lottery when the expected outcome is favorable. Studies in experimental economics have repeatedly shown, however, that human rationality can be affected by judgment errors, meaning that decision-making is not always optimal with regard to the maximization of profit [6]. For example, individuals do not necessarily exhibit the same pattern of response according to the domain of risk; some may be risk seekers when they practice a dangerous sport, and altogether be risk adverse when buying an insurance against some very unlikely disasters. Another usual departure from rationality is the pattern of loss aversion, where losses affect individuals more than gains; to equate the psychological weight of a loss, their potential gain must be worth two times the loss [7] and this coefficient was found stable in several experimental setups [7], [8]. Individuals' decisions can be strongly affected by loss aversion and by other judgment errors in risky contexts. Another judgment error is probability distortion. For instance, individual over-evaluate the probability of winning in state lotteries or, in the same vein, they underweight losing probabilities, possibly because they are optimistic. Overweighting losses may appear little rational at first sight, but it makes sense in critical situations. “It is better to be safe and believe that a tiger is nearby rather than believe that no tiger is nearby and be sorry if it turns out you're wrong” (from [9], p 62). This “better be safe than sorry” strategy likely lies on rules of thumb which maximize survival rather than immediate benefits [9]. Thus, while judgment errors may not be optimal in term of immediate maximization of resources, they can represent a selected trait that once had an important positive survival value. Using a rule of thumb may be especially relevant for children who may fail to understand the risky nature of their surroundings. It could prevail when rational evaluation of surroundings is not yet fully matured. Little is known about the emergence of judgment errors in children, but if they represent a remnant of a once survival mechanisms we should expect that they appear early in childhood.

Attitudes towards risk are known to develop with age [10] and a common observation is that children are greater risk takers than adults [10]–[13]. Children are arguably less experienced regarding the negative consequences of a risky decision. They may also lack the cognitive maturation required for accurately evaluating risks [10]. The study of risk sensitivity in children requires a two-fold approach. First, we need to evaluate the rationality of their decision-making in a risky context. Second, if decisions are not always rational, we need to decipher whether they are substantiated by judgment errors and to what extent. To this aim we need to consider the models of decision making elaborated in classical economy for adults. The Expected Utility Theory (EUT), introduced by von Neumann and Morgenstern [14], states that individuals choose among risky prospects by comparing their expected utility values, based on their respective probabilities (see [15]). According to EUT, a rational individual who is offered a choice between a lottery and a certain amount is supposed to choose the option maximizing his expected utility. Moreover, if children behave as rational decision-makers, we should expect that they do not distort probabilities, and that they do not exhibit loss aversion. Adults are generally rational decision-makers [16], but their decisions sometimes deviate from EUT predictions [6], [17]. For instance, human buy lottery tickets although, according to EUT, the expected value of the game is negative. In this context, they overweigh the probability of unlikely outcomes [6], [7], [18]. Cumulative Prospect Theory (CPT) was developed to account for these judgment errors. CPT differs from EUT in two ways. On the one hand, value is assigned to outcomes relative to a reference point rather than to final wealth [7] so that they are evaluated in terms of gains and losses and losses are weighted more heavily than gains. On the second hand, the model includes a non linear probability weighting function, which takes into account the fact that individuals distort probabilities: overweighting chances in the domain of losses, underweighting them in the domain of gains. An understanding of risk similar to the adult is found at around 11–12 years of age [10]–[12], [19], and several errors in judgment have been reported in adolescents [20]–[22]. While adolescent have the mean to evaluate the risk inherent to a given context, their decisions are strongly affected by a heightened emotional responsiveness that is typically aroused in situation of risk or when gains are at stakes. This affective decision-making [23] is thought to frequently overrule a more rational evaluation, which would explain risk-seeking conducts at youth age. In that respect, adolescents represent a “special case” in the study of attitudes towards risk.

Children as young as 2 years of age can enter in exchanges with experimenters [24], and from 4–5 years they display a good intuitive understanding of probability concepts in risky decision-making [25]–[27]. Harbaugh et al. [10] tested 5- to 13-year-old children in a game where they could choose between a sure outcome (gain or loss) and a lottery with the same expected value. Winning led to obtain tokens that could be used afterwards to buy toys. They found that children underweighted low-probability events and overweighed high-probability ones. However, these results cannot be generalized in the context of EUT or CPT since children based their value judgment on secondary reinforcement and not on a direct evaluation of utility. Besides, the occurrence of other judgment errors such as loss aversion and their strength was not estimated.

In this study we tested the attitude towards risk of children aged between 3 and 9 years in a simple choice task between a lottery and a certain outcome. Children were first endowed with an initial piece of cookie (the certain outcome) that they could exchange for the content of one cup chosen at random among six visible (the lottery). The food rewards contained in the cups could be larger, equivalent, or smaller in size than the initial endowment making it possible to evaluate directly the utility of the outcome. We manipulated the chances to win or lose and recorded the children choices. By confronting their choices to the predictions from either EUT which assumes rational decision-making, or from CPT which assumes probability distortion and loss aversion, we aimed at deciphering 1) if children decision-making is fully rational in a risky context (i.e. allowing them to maximize their expected utility as predicted by the EUT model), and if not 2) whether their judgment is affected by known judgment errors and how early they occur. If judgment errors indeed possess a survival value, and once compensated for a slow maturation of the brain, they should be detectable early on in children.

## Methods

### Ethics Statement

Ethical authorization to work with children was given by the University of St-Andrews ethics committee, UTREC (reference n°PS5528). Parents were given a letter describing the general purpose of the study and written parental consent was required for children to participate in the tests. Participation was on an unpaid, voluntary basis, but children kept the sweets that they won during the testing session. A video camera recorded the session after parental written consent had been obtained.

### Participants and conditions

A sample of 288 children was divided into six age-groups: 3 years old (mean age ± SEM = 42.3±2.2 months), 4 years old (mean age = 54.3±4.2), 5 years old (mean age = 65.5±1.9), 6 years old (mean age = 78.0±3.4), 7 years old (mean age = 89.3±3.6) and 8 years old (mean age = 100.0±4.0). Equal numbers of girls and boys were tested in each of groups. Twelve additional children whose parents had given consent were tested, but were excluded from the dataset analyzed for being outside of the appropriate age ranges, or for not paying attention to the tests. Participants were European, with English as their first language. The experiment took place at the Living Links to Human Evolution' Research Centre in Edinburgh zoo. Children were recruited upon their visit to the “Budongo trail”.

**Figure 1:**
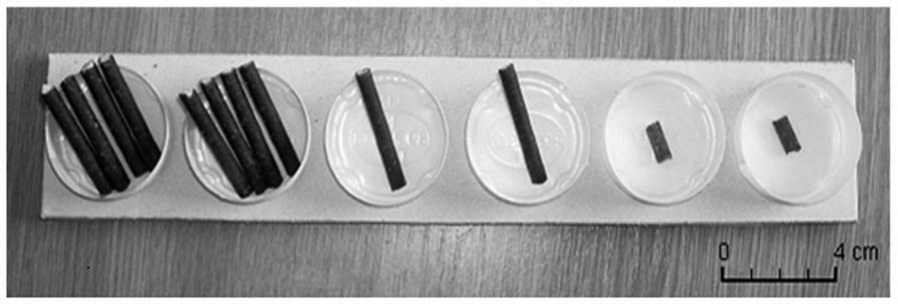
Six plastic cups containing pieces of cookies of various dimensions: two pieces of cookie of 4×2×0.5 cm (sequence of four 4×0.5×0.5 cm - left position), two pieces of cookie of 4×0.5×0.5 cm (middle position) and two pieces of cookie of 1×0.5×0.5 cm (right position) corresponding to the combination of rewards # 4.

Tests were conducted in a small area (2.5×2 m) limited by four occluders allowing an entire visual seclusion from public. Children were individually tested while seated on a chair or on their parent's lap in front of a square table (1×1 m). The apparatus consisted of six aligned plastic cups containing pieces of cookies of various dimensions (Figure 1).

**Table 1:** Number (#) and content of cups for each combination of rewards.

Combinations of rewards													
	#	Content of cups					#	Content of cups					
0	L	L	L	L	L	L							
1	L	L	L	L	M	M	6	L	M	M	M	M	M
2	L	L	L	L	S	S	7	L	M	M	S	S	S
3	L	L	L	M	S	S	8	L	M	S	S	S	S
4	L	L	M	M	S	S	9	L	S	S	S	S	S
5	L	L	S	S	S	S	10	S	S	S	S	S	S

S (Small) indicates pieces of cookie of dimension 1×0.5×0.5 cm, M (Medium) indicates pieces of cookie of 4×0.5×0.5 cm, and L (Large) indicates pieces of cookie of 4×2×0.5 cm (sequence of four 4×0.5×0.5 cm pieces of cookie).

### Exchange procedure

The experimenter was unfamiliar to the children. Before testing, the experimenter introduced herself to the child to put them at ease. Only one parent was allowed to stay with the child during testing. Parents were instructed not to interfere by initiating communication or interactions during testing; if a child sought interaction with parents, they were asked to draw the child's attention back to the experimenter without directing her/his response or give any hints of what s/he was supposed to do. If the child wanted to interact with the experimenter, she repeated the instructions and encouraged her/him to redirect her/his attention to the task. During testing children were seated on a chair across from the experimenter.

The experiment lasted approximately 20 minutes. During the training phase, each child was asked to indicate a preference for either a small piece of cookie of dimension 1×0.5×0.5 cm or a larger piece of cookie of dimension 4×0.5×0.5 cm. Each child selected the larger reward. Then, the child was given the small piece of cookie, and was invited to return it to obtain the larger one. If the child exchanged successfully, the test started. If s/he failed, the request was repeated twice. If nothing happened after 5 s, testing ended. We did not control for the level of hunger of children. However, subjects were given a plastic bag, and they were informed that they could save for later the cookie that they received.

In each trial the experimenter first presented in one hand the initial piece of cookie of 4×0.5×0.5 cm, and in the other six aligned plastic cups containing pieces of cookie of various dimensions: 1×0.5×0.5 cm (small size), 4×0.5×0.5 cm (medium size) and 4×2×0.5 cm (large size - sequence of four 4×0.5×0.5 cm pieces of cookies) (Figure 1). The experimenter gave the initial medium piece of cookie to the child, and held out her empty hand offering the child the chance to give the initial item back “Here is a piece of cookie and here are others cookies. Do you prefer to keep the cookie or do you want to swap it to have one of these one?”. If the child returned it, s/he received the content of one cup chosen randomly. (randomization was implemented prior to the session using a software, www.randomizer.org). If the child chose to keep the initial item, the experimenter ended the trial, allowing the child to consume the initial item or store it into a bag (the child was told that the bags could be used to store the cookies so that the child could take them with him after the game). Whatever the choice of the child, the experimenter proceeded with the next trial.

The probability to lose and to gain was manipulated via 11 combinations of two trials each (Table 1). A first half of children were run in Condition A presenting a step by step decrease in the chances to win from combinations # 0 to # 10 then a step by step increase in the chances to win from combinations # 10 to # 0. The other half was run in Condition B presenting first gradually increasing chances to win then decreasing chances.

### Analysis procedure and theoretical predictions

Economic theoretical models require individuals to respect first-order stochastic dominance which translate in our study by always exchanging when 100% chances of winning and never exchanging when 100% chances of losing. In our dataset, 24.7% of the children always accepted to exchange at the combination # 0 (no loss) and always refused at the combination # 10 (no gain). Because the percentage of return at each combination between these children and the whole population was similar according to age, we further included the data of all the tested children in the models.

Von Neumann and Morgenstern [14] introduced the Expected Utility Theory (EUT) stating individual preferences with a mathematical function named as “utility function”. Under EUT, we assume that subjects evaluate values of outcomes in terms of utility, measured by the volume of each piece of cookie, because they are consumption amounts [28], [29]. The item volume of options *x_i_* are weighted by their respective probabilities *p_i_*, and compared by the individuals to determine their choice. Let *x = ((x_i_, p_i_),i = 1,…,n)* denote a lottery where *x_i_* is the *i*-th outcome, and *p_i_* the corresponding probability. Rational individual who is offered a choice between the lottery *x* and a certain amount *W* is supposed to choose the option with the higher expected value maximizing their expected utility and to keep the certain amount if:
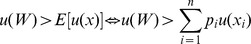
As for the utility function we chose the power function *u(y) = y^δ^, µ* y, because it allows to derive risk preferences of individuals. In this function, *y* is the quantity of item and *δ* is the risk aversion parameter. Evaluating each item value *y* is a straightforward way to measure the item volume. Indeed, smaller pieces of cookies of 1×0.5×0.5 cm were valued 0.25, pieces of cookie of 4×0.5×0.5 cm were valued 1, and larger pieces of cookie 4×2×0.5 cm were valued 4. As children could obtain only one piece of cookie among the six offered, the evaluation of each combination of rewards is also straightforward (Table 2). For instance, the combination # 3 containing 2 small, 1 medium and 3 large rewards was evaluated E[u(#3)] = (2/6×0.25*^δ^*)+(1/6×1*^δ^*)+(3/6×4*^δ^*) in EUT.

**Table 2:** Evaluation of each combination of rewards following the Expected Utility Theory (*δ* is the risk aversion parameter), with δ = 1 (neutrality to risk).

#	EUT	δ = 1	EUT evaluation (δ = 1)
0	4^δ^.(6/6)	4	**EU>certain amount**
1	4^δ^.(4/6)+1^δ^.(2/6)	3	
2	4^δ^.(4/6)+0.25^δ^.(2/6)	2.75	
3	4^δ^.(3/6)+1^δ^.(1/6)+0.25^δ^.(2/6)	2.25	
4	4^δ^.(2/6)+1^δ^.(2/6)+0.25^δ^.(2/6)	1.75	
5	4^δ^.(2/6)+0.25^δ^.(4/6)	1.5	
6	4^δ^.(1/6)+1^δ^.(5/6)	1.5	
7	4^δ^.(1/6)+1^δ^.(2/6)+0.25^δ^.(3/6)	1.125	
8	4^δ^.(1/6)+1^δ^.(1/6)+0.25^δ^.(4/6)	1	**EU = certain amount**
9	4^δ^.(1/6)+0.25^δ^.(5/6)	0.875	**EU<certain amount**
10	0.25^δ^.(6/6)	0.25	

From combination # 0 to # 7, the expected utility is superior to the certain amount, and children should exchange. For combination # 8, the expected utility is equal to the certain amount. For combinations # 9 and # 10, the expected utility is inferior to the certain amount, thus children should not exchange.

In each trial the experimenter offered the medium item, and children had to choose between keeping it or giving it back in order to obtain one cup of rewards. According to EUT children should prefer the gamble to the medium item if the expected utility of the gamble exceeded the utility of the certain outcome (Table 2). For instance, in # 3 the gamble was preferred if [(#3)]>(1)*^δ^*. Under the simpler assumption that children are risk neutral (δ = 1), they should exchange rather than keep the medium item from combination # 0 to # 8 when the expected utility was equal to or higher than the utility of the certain initial amount (Table 2). Conversely, children should refuse to exchange in the last combinations (# 9 and # 10) because the expected utility is lower than the utility of the certain outcome. If subjects are not risk neutral (δ≠1), we need to assess a risk aversion parameter δ. Children are risk averse (δ<1) when, between two options with identical expected values, they prefer the safer option, and risk-seeking (δ>1) when they choose the risky option [30]. Detecting at which combination children become indifferent between the risky and the certain item can be used to infer the risk aversion parameter values.

Under Cumulative Prospect Theory (CPT), subjects evaluate the opportunity to play the lottery *x = ((x_i_, p_i_),i = 1,…,n)* by computing a valuation function *V(x)* defined as follows:
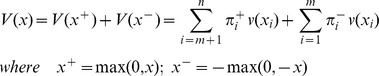
The first element of CPT is to define *x* in terms of net gains and losses, and to rank the outcomes of the lottery in increasing order. The evaluation function is defined over these two domains with different decisions weights 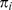 and a specific value function *v(.)*. The value function is analogous to the utility function of EUT but is defined differently over gains and losses:
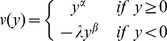
with λ>1 (loss aversion) and 0<*α, β*≤1 (diminishing sensitivity)

The value function is generally concave on gains, convex on losses, and kinked at 0. The loss aversion parameter λ indicates that subjects are loss averse if λ>1, which means that in any choice where a loss of *k* is at stake, subjects accept the bet if the net potential gain is higher than λ times *k*. Tversky and Kahneman [8] experimentally identified a median value of λ = 2.25, indicating pronounced loss aversion. In our study this value would mean that children accepted to lose a medium piece of cookie if s/he could gain a piece of cookie with a size 2.25 times the medium piece of cookie.

**Figure 2:**
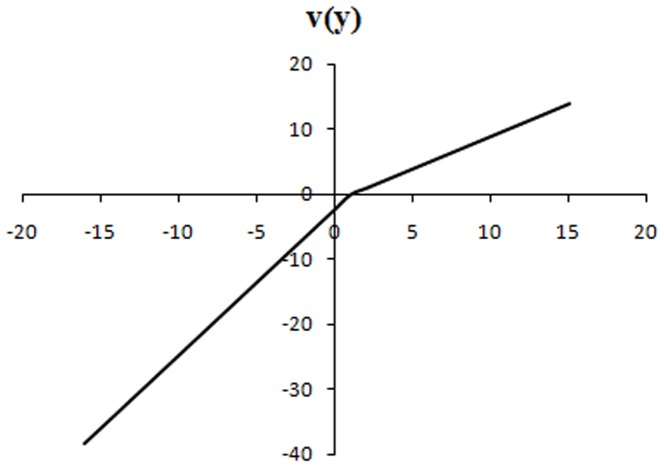
Kahneman and Tversky evaluation function for α = β = 1, λ = 2.25, and a reference point of 1.

Here, gains and losses were amounts of cookies that were directly consumed by children in excess or less than the reference consumption amount 1. To simplify, we used α = β = 1 (for piecewise linear functions, Figure 2). For instance, for children using CPT in their choice, combination # 3 was perceived as an opportunity to realize 3 gains (3 large instead of 3 medium items), each evaluated 3 (4 - 1), and to incur 2 losses (2 small instead of 2 medium items), each evaluated −0.75 (0.25–1).

The second element explaining the choice of children using CPT in decision-making is the weighting parameter. Individuals use weights of outcomes instead of probabilities, represented by a non-linear function 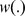 that is defined separately on the cumulative probability distribution of gains (+) and losses (−):
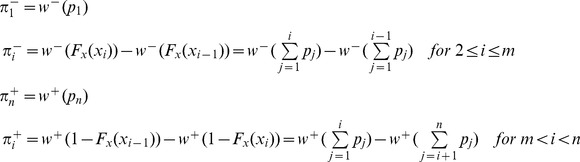
where *F_x_* is the cumulative distribution function of lottery *x*. The probability weighting function put forward in the literature is generally inverse S-shaped. To simplify, we used *w^+^(.) = w^−^(.) = w (.)* and made our computations with the Tversky and Kahneman [8] probability weighting function:
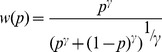
where γ is the probability distortion parameter, w(0) = 0 and w(1) = 1

**Figure 3:**
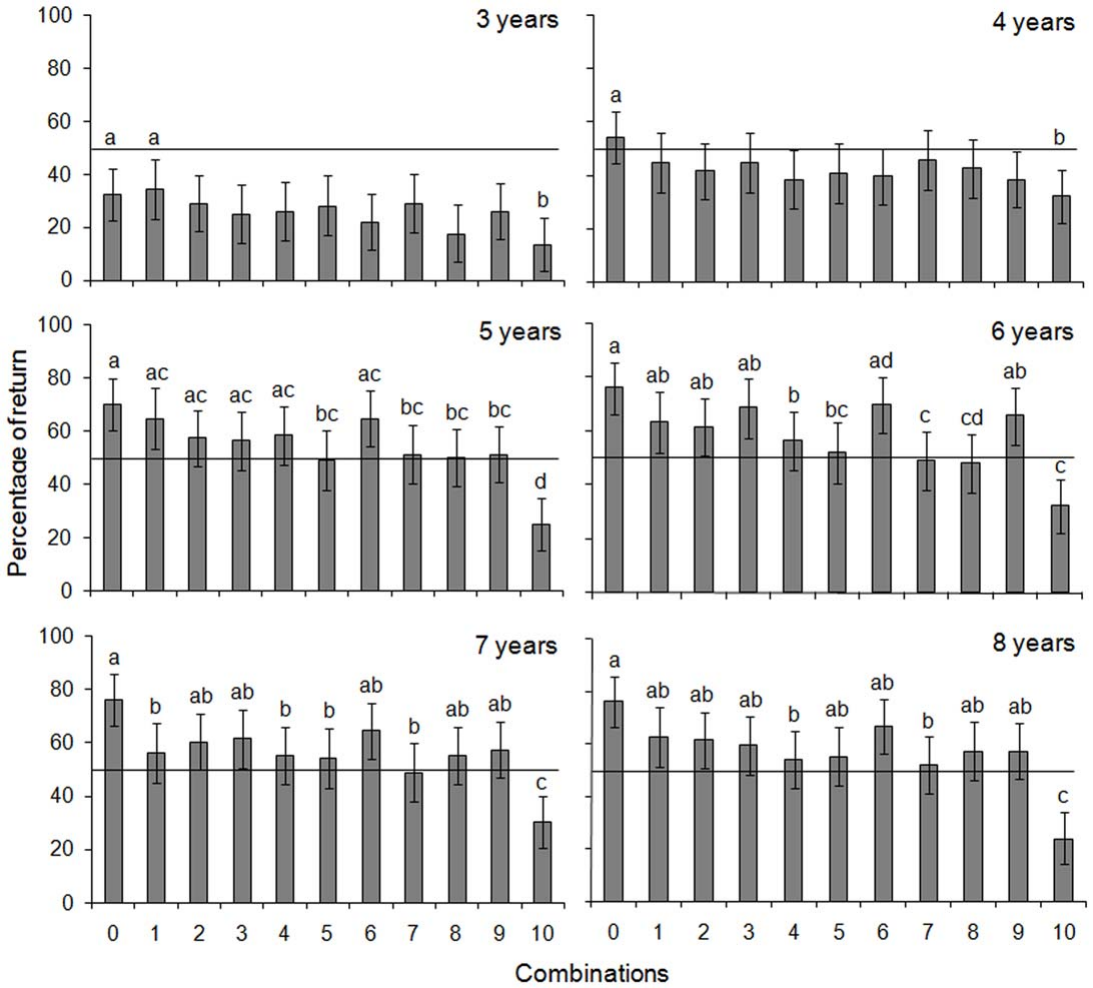
Percentage of returned items for each age according to the combination of rewards. The bar indicates the threshold of 50% of exchange. Combinations with no common letters differ significantly at *p*<.05.

This specific probability weighting function takes the shape of an inverse S if 0<γ<1, which is a common characteristic of all weighting functions [31]. In this function, γ both controls for the level of the inflexion point and for the curvature. Tversky and Kahneman [8], Camerer and Ho [32] and Wu and Gonzalez [33] have estimated the weighting function to a value of 0.56, 0.61 and 0.71, respectively. Figure 3a gives an example with a value γ = 0.6 estimated by Tversky and Kahneman, meaning that subjects perceive low probabilities (under 0.35) higher and high probabilities (over 0.35) lower than their actual value. In adults, studies showed risk-seeking over small-probability gains and high-probability losses, and risk-aversion over high-probability gains and small-probability losses [8].

The evaluation of each combination deeply depends on values chosen for γ and λ. Table 3 presents CPT evaluations of all combinations of rewards with no probability distortion, i.e. γ = 1, and with no loss aversion, i.e. λ = 1. For instance, for γ = 1 (no probability distortion as *w(p) = p*) and λ = 2.25 (loss aversion), we would get V(# 3) = 1.88 which would indicate that, according to CPT, children should accept to exchange the initial item. A negative value would indicate that children should keep the initial item.

**Table 3:** Evaluation of each combination of rewards following the Cumulative Prospect Theory (without probability distortion and loss aversion).

	CPT	CPT
#	no probability distortion: γ = 1	no loss aversion: λ = 1
0	0	6.(1-w(1))
1	3	6.(1-w(1/3))
2	4-0.5λ	6.(1-w(1/3))-3.375.w(1/3)
3	3-0.5λ	6.(1-w(1/2))-3.375.w(1/3)
4	2-0.5λ	6.(1-w(2/3))-3.375.w(1/3)
5	2-λ	6.(1-w(2/3))-3.375.w(2/3)
6	1	6.(1-w(5/6))
7	1-0.75λ	6.(1-w(5/6))-3.375.w(1/2)
8	1-λ	6.(1-w(5/6))-3.375.w(2/3)
9	1-1.25λ	6.(1-w(5/6))-3.375.w(5/6)
10	1-1.5λ	6.(1-w(1))-3.375.w(1)

## Results

The decision to exchange was affected by age (GLM, z = 2.6, *p*<0.001), the younger children exchanging less than the older children (Figure 3). This was particularly true for the 3- and 4-year-olds (percentage of return = 25.8% and 42.2%) compared to other groups (5 years: 54.3%; 6 years: 58.2%; 7 years: 56.3% and 8 years: 56.9%; Table 4). Children exchanged more (GLM, z = 1.4, *p*<0.001) for “winning only” (64.1%, combination # 0) and “zero chances to lose” combinations (54.5%, combination # 6) than for “losing only” combination (26.2%; combination # 10). A combined effect of age and combination affected their responses. Before 5 years, children discriminated only between “no chances to win” (combination # 10) and “no loss” (combinations # 0 and/or # 1). Above 5 years, all age groups discriminated between “no loss” (combination # 0) and “no win” (combination # 1), and both these combinations were significantly discriminated from most of the others (Table 5). With respect to conditions – condition A with increasing chances to win then decreasing chances, and condition B with decreasing chances then increasing – we observed a framing effect; children starting with winning combinations exchanged more often than children starting with losing combinations (GLM, z = 2.2, *p*<0.05). Exchange rates were low for combinations with highest probability of loss (# 9 and # 10) regardless of the condition (GLM interaction condition x combination, z = 1.2, *p*<0.001).

**Table 4:** Comparisons of the mean percentage of return between the different age-groups using Tukey HSD pairwise comparison post-hoc tests.

	Tukey HSD pairwise comparison test	
age	Z value	p
3-4	3.37	0.13
3-5	5.94	<0.001
3-6	6.96	<0.001
3-7	6.40	<0.001
3-8	6.30	<0.001
4-5	2.59	<0.05
4-6	3.61	<0.05
4-7	3.03	<0.01
4-8	2.96	<0.01
5-6	1.03	0.91
5-7	0.42	0.99
5-8	0.39	0.99
6-7	−0.62	0.99
6-8	−0.64	0.98
7-8	−0.03	1

**Table 5:** Tukey HSD pairwise comparison post-hoc tests for combinations significantly differing by the mean percentage of return.

	3 years		4 years		5 years		6 years		7 years		8 years	
combinations	Z value	p	Z value	p	Z value	p	Z value	p	Z value	p	Z value	p
1-0									−3.29	<0.05		
4-0							−3.43	<0.05	−3.30	<0.05	−3.29	<0.05
5-0					−3.58	<0.05	−4.14	<0.05	−3.30	<0.05		
7-0					−3.24	<0.05	−4.42	<0.001	−4.27	<0.001	−3.58	<0.05
8-0					−3.41	<0.05	−4.56	<0.001				
9-0					−3.24	<0.05						
10-0	−3.63	<0.05	−4.05	<0.01	−7.19	<0.001	−6.56	<0.001	−6.50	<0.001	−7.13	<0.001
10-1	−3.97	<0.01			−6.46	<0.001	−4.90	<0.001	−3.63	<0.05	−5.47	<0.001
10-2					−5.40	<0.001	−4.16	<0.001	−4.47	<0.001	−5.33	<0.001
10-3					−5.24	<0.001	−5.33	<0.001	−4.61	<0.001	−5.07	<0.001
10-4					−5.55	<0.001	−3.57	<0.05	−3.63	<0.05	−4.39	<0.001
10-5					−4.13	<0.01			−3.63	<0.05	−4.53	<0.001
7-6							−3.31	<0.05				
8-6							−3.46	<0.05				
10-6					−6.46	<0.001	−5.61	<0.001	−4.75	<0.001	−6.00	<0.001
10-7					−4.45	<0.001					−4.12	<0.001
10-8					−4.29	<0.001			−3.77	<0.01	−4.80	<0.001
10-9					−4.45	<0.001	−4.89	<0.001	−3.91	<0.01	−4.80	<0.001

We further assessed the effect of age, probability of winning P*_G_*, probability of losing P*_L_*, and variables resulting from previous outcomes (O*_P_*: outcome received at the previous trial, O*_S_*: cumulative outcome since the start of the mid-condition, O*_CUM_*: cumulative outcome since the start of the testing session) on the response of children. There was no significant effect of outcomes on the percentage of exchange (GLM, O*_P_*: z = 0.7, *p* = 0.18; O*_S_*: z = 0.1, *p* = 0.99; O*_CUM_*: z = 0.1, *p* = 0.94). Age (GLM, z = 5.8, *p*<0.001), and probabilities of winning (GLM, z = 4.5, *p*<0.001) or losing (GLM, z = −6.7, *p*<0.001) significantly affected responses (Table 6). The probability of losing affected the responses of the 5-, 6- and 7-year-olds more than any other factor. The 8-year-old children took both the probability of winning and the probability of losing into account. A similar trend was observed in 7-year-olds. The percentage or return decreased with decreasing expected utility and children exchanged significantly less often when the expected utility was inferior to the certain amount (i.e. 1, for the combinations # 9 and # 10).

**Table 6:** Influence of the probability of losing P*_L_* (getting a small piece of cookie in this trial), the probability of gaining P*_G_* (getting a large piece of cookie in this trial), and the interaction P*_L_* x P*_G_* on the exchange behaviors of children.

Variables	3 years	4 years	5 years	6 years	7 years	8 years
P*_L_*	z = −1.081	z = −0.345	z = −4.267	z = −3.303	z = −2.554	z = −3.777
	(p = 0.28)	(p = 0.73)	(p<2e−5)	(p<9e−4)	(p<0.05)	(p<2e−4)
P*_G_*	z = 1.407	z = 1.499	z = 0.305	z = 1.370	z = 1.670	z = 0.991
	(p = 0.16)	(p = 0.13)	(p = 0.76)	(p = 0.17)	(p = 0.10)	(p = 0.32)
P_L_*P*_G_*	z = 0.683	z = −0.364	z = 1.414	z = 0.861	z = 1.866	z = 1.994
	(p = 0.50)	(p = 0.72)	(p = 0.16)	(p = 0.39)	(p = 0.06)	(p<0.05)

In view of the previous results, we conducted analyses on the three age categories: 3–4 years, 5–6 years, and 7–8 years to test whether their response fitted with EUT. We compared the frequency of choices of the lottery against 50%, i.e. the frequency obtained if children decided randomly whether to exchange or not. In accordance with EUT the percentage of returned items for each age decreased from the combination of rewards # 0 to # 10. All children presented a percentage of return significantly smaller than 50% only for combination # 10 (Table 7), which was not consistent with EUT predicting that, under risk neutrality (δ = 1), children should not exchange for combinations # 9 and # 10 (Table 2). Given that under EUT, risk neutrality could not explain the behaviour of children, we inferred a risk aversion parameter (δ) by detecting the combination at which the percentage of return became significantly lower than 50%. The low return rates of 3–4 years for most combinations (inferior to 50%) did not allow finding this value. For children aged 5–6 and 7–8 years, the certain loss combination (# 10) was the one for which the percentage dropped under 50% (Table 7); we found δ≥1.17 which is >1 and indicates that they were risk-seekers. However, risk-seeking children (δ>1) should also exchange at a higher rate in combination # 5 than in combination # 6 (risk-neutral children, δ = 1, should exchange at the same rate for combinations # 5 and # 6 and risk-averse children, δ<1, should exchange at a lower rate in combination # 5 than in combination # 6, Table 2). Contrary to this prediction, children aged 5–6 and 7–8 years exchanged significantly more often for combination # 6 than # 5 (Student's *t*-*test*, 5–6 years: t_47_ = −3.67, *p*<0.01; 7–8 years: t_47_ = −1.97, *p*<0.05, Table 7). Under EUT, no risk parameter value can explain both refusal to exchange for combination # 10, and preference to exchange for combination # 6 compared to # 5. This final result strongly indicates that children decisions could not be fully accounted for in a EUT framework.

**Table 7:** Student's t-tests on the percentage of return for each combination.

Combinations	Number of cups			3–4 years		5–6 years		7–8 years		mean % of return
#	Large	Medium	Small	t	p	t	p	t	p	
0	6	0	0	−1.73	0.09	7.09	<0.001	7.83	<0.001	64.07
1	4	2	0	−2.31	*<0.05*	4.23	<0.001	2.21	<0.05	54.34
2	4	0	2	−3.78	*<0.001*	2.44	<0.05	3.14	<0.01	51.90
3	3	1	2	−3.85	*<0.001*	2.92	<0.01	2.75	<0.01	52.30
4	2	2	2	−4.58	*<0.001*	1.85	0.07	1.18	0.25	48.09
5	2	0	4	−3.86	*<0.001*	−0.15	0.88*	1.16	0.25*	46.52
6	1	5	0	−5.05	*<0.001*	4.80	<0.001*	4.32	<0.001*	54.53
7	1	2	3	−3.29	*<0.01*	0	1	0.13	0.90	46.01
8	1	1	4	−4.78	*<0.001*	−0.31	0.76	1.73	0.09	45.13
9	1	0	5	−4.58	*<0.001*	2.42	<0.05**	1.96	<0.05**	49.31
10	0	0	6	−7.97	*<0.001*	−6.42	*<0.001* **	−6.18	*<0.001* **	26.22
mean % of return				33.95		56.34		56.62		
				(3 years = 25.76; 4 years = 42.15)		(5 years = 54.27; 6 years = 58.42)		(7 years = 56.34; 8 years = 56.91)		

Italic values indicate that the mean number of return for a combination is *significantly* smaller than 50%; non-italic values indicate that the mean number of return for a combination is *significantly* higher than 50%.

** : combinations for which children accepted to exchange for a specific combination, but refused for the following one;

* : combinations offering the same evaluation in the EUT at which children should exchange at the same rate.

**Figure 4:**
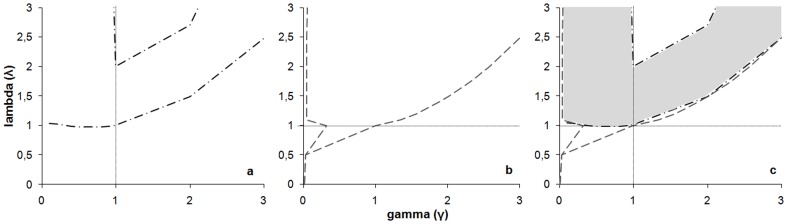
Estimation of the loss aversion parameter λ, and the probability distortion parameter γ: **a**. Under the assumption of no probability distortion (fixing arbitrarily γ = 1, grey continuous line), 1<λ≤2; **b**. Under the assumption of no loss aversion (fixing arbitrarily λ = 1, grey continuous line), 0.32<γ<1; **c**. Acceptable values for both loss aversion and probability distortion parameters (grey area).

To account for children's deviation from the rational choice predicted by EUT, we investigated whether their response can be better explained by another theoretical model, the Cumulative Prospect Theory (CPT, Table 3). In this model, value is assigned to gains and losses relative to a reference point rather than to final wealth [7], [8]. Choices can depend on the level of the loss aversion parameter (λ) and the level (γ) of the weighting function parameter, i.e. how children distort probabilities. Although the utility of combinations # 5 and # 6 is equivalent, the presence of the potential for loss (presence of small pieces of cookies) in combination # 5, and its absence in combination # 6, may explain the responses of children. To examine this hypothesis, we first made the assumption of no probability distortion (fixing arbitrarily γ = 1), and we determined the corresponding level of the loss aversion parameter (λ) based on the return rate of the combinations # 5 and # 6. The return rate of children aged 5–6 and 7–8 was higher than 50% for combination # 5 and # 6 (albeit not significant for combination # 5, Table 7), thus we search the value of λ for which children accepted the gamble for both combinations, and for which they exchanged more for combination # 6 than # 5. We found 1<λ≤2 for both age categories (Figure 4a); this indicates that children aged over 5 were highly loss averse: when a loss was at stake, subjects accepted to exchange if the net potential gain was higher than at least 1 to 2 times the loss.

**Figure 5:**
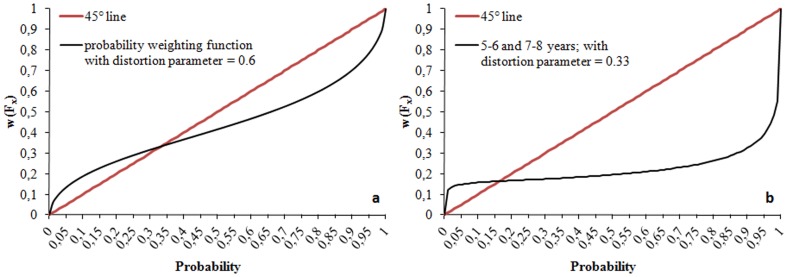
Probability weighting function a. for γ = 0.6 (Kahneman and Tversky). The inflexion point is close to a probability of 0.35; individuals perceive low probabilities under 0.35 higher than their actual value and high probabilities over 0.35 lower than their actual value. **b. for children aged over 5 years** (0.32<γ<1). For γ = 0.33, the inflexion point is close to a probability of 0.15; individuals perceive low probabilities under 0.15 higher than their actual value and high probabilities over 0.15 lower than their actual value.

If we make the assumption of no loss aversion (fixing arbitrarily λ = 1), we can employ the Kahneman and Tversky's probability weighting function in order to find γ, the probability distortion parameter. For both age groups, we determined the value of γ for which children accepted the gamble for combinations # 5 and # 6, but exchanged more for combination # 6 than # 5. We found 0.32<γ<1 for both age groups (Figure 4b). This value points at a strong probability distortion. The function takes the shape of an inverse S-shaped with an inflexion point close to a probability of 0.15; individuals perceived low probabilities under 0.15 higher than their actual value, and high probabilities over 0.15 lower than their actual value (Figure 5b). However, for these values of γ we cannot explain why children rejected the gamble for combination # 10.

The loss aversion parameter λ and the probability distortion parameter γ can also be assessed together. For both categories, we determined the values of λ and γ based on the conditions previously used, i.e. the return rate of combinations # 5 and # 6, and the combination after which children rejected the gamble. We found that values of γ were acceptable only when λ>1 (Figure 4c), indicating that loss aversion was critical in the choices of 5–6 and 7–8 years old. For the probability distortion parameter, all values of γ were acceptable (< or >1; Figure 4c). For instance, λ = 2.5 and γ = 0.6 meant that children were loss averse, and overweighed low-probability events while underweighting high-probability ones. As another example for acceptable values, λ = 1.5 and γ = 1.3 meant that children were loss averse, and underweighted low-probability events while overweighting high-probability ones. These results on loss aversion hold if the probability weighting function is either convex (or concave) for gains, and concave (or convex) for losses. Therefore, the non-linearity of the value function (loss aversion) override the non-linearity of the weighting function, making loss aversion the main discriminatory criterion in choices of children aged over 5.

## Discussion

All children engaged in a trading game providing a typical risky economic situation. Children aged 3 to 4 years old displayed an exchange rate inferior to 50%, so we could not identify risk-related biases in their decision-making. At this age, children display good performances in delay-of-gratification tasks, including the exchange task [24], [34]; thus, the ability to control impulsivity, i.e. keeping the initial endowment instead of eating it, was not a limiting factor in this study. Lesser abilities in probabilistic evaluation may explain this failure as they only discriminated between the no gain and no loss combinations. In everyday life children commonly encounter binary choices (“you will have or you won't have a dessert”), but are seldom submitted to choices offering probabilities (“you have a one in five chance to have a dessert”). Failure in adapting their return to combinations may also be due to the use of heuristics, i.e. intuitive judgment; for example they may have used a rule consisting in summing the content of the six cups (“I see a lot of cookies, I try”).

Children aged 5–6 years were able to adapt their return according to the combinations of rewards. Responses were mainly based on the probability of losing. This finding is consistent with the development of probability estimation by age 5 [25], [27], [35], [36]. Although probably incomplete [37], [38], the understanding of probabilistic rules in 5-year-old children allowed them to attempt optimizing their benefits. Better performances were observed in children aged 7–8 years, as they were more discriminative with the combinations. Still, the decision was not always rational given that some children kept on exchanging for the “no win” combination, and refused for the “no loss” combination.

Our results show that decision-making was affected by several judgment errors. We detected a framing effect; children starting with high probabilities of gain exchanged more than children starting with low probabilities of gain. Variations in framing are known to affect significantly decisions and risk-aversion in adults [39], [40]. Individuals are risk-averse when presented with value-increasing options, but more risk-taking when faced with decreasing values [41]–[43]. Recent studies have reported that behavioral responses to framing may vary according to the set-up used [40]. For example, high incentives to win can have more impact on risk aversion than low incentives. Future research will have to compare the framing effects detected in children with those found in adults.

When confronting data to the expected utility model, we found that children aged over 5 appeared to behave almost consistently with EUT model with a risk-seeking attitude. However some results could not be explained in this framework. Instead, the definition of rationality as in EUT should be alleviated and CPT could explain better the errors observed among children decisions. Specifically, implementing a loss aversion parameter strongly explained deviations from rational behavior. The acceptable values of loss aversion parameter is comprised between 1 and 2, which is close (although inferior) to Kahneman and Tversky's experimentally validated value of 2.25 for adults and adolescents [7], [8]. This means that, facing a risky decision, children accept to exchange if the net potential gain is higher than at least one to two times the amount of the loss. Adult's studies suggest that loss aversion can depend on the initial wealth and the level of trust and education [44]. Finding loss aversion in young children supports the notion that this pattern is not solely depending on such environmental factors. Further studies involving adults with the same paradigm should help decipher whether the difference in value of the loss aversion parameter reflects (or not) a stronger aversion to loss in adult compare to children. A second parameter known to affect decision-making in risky situations is probability distortion. It is often reported for adults in circumstances involving extreme probabilities such as state lotteries [45] and was reported in children by Harbaugh et al. [10]. Here, we also found probability distortion with children overweighting or underweighting probabilities. However, we could evaluate its relative impact compared to the loss aversion effect. We found that the value function (loss aversion) overrode the nonlinearity of the weighting function. This makes loss aversion the main pattern explaining children's decision-making.

From a neuropsychological point of view, the general improvement in rational decision-making between 3 and 8 is in agreement with the development of several brain functions involved in the control of impulsivity and reward valuation [23]. This development is non linear as the brain circuits supporting those functions mature at different speed. Self-regulatory mechanisms involved in the control of impulsivity [46] are related to the maturation of the prefrontal cortex that is the latest structure to be fully mature – this occurs at around 20 years. Affective decision-making, i.e. the fact that decision is partially governed by emotions depends among other structures on the maturation of the nuclei accubens, and reach a pick of responsiveness at adolescence [46]. The adolescents experience the emotions associated to risk, and are attracted by gains more strongly than younger children or even adults, which explains the observation that they can be more risk seeking. Eight-year-old children have better control of impulsivity than younger ones, yet they are not at an age where affective decision-making will overrule their rational evaluation, thus the improved rationality in their decisions compared to younger children appears consistent with what would be expected.

The occurrence of adult-like loss aversion in children as young as 5 confirms that judgment errors appear early, suggesting that it is deeply rooted in human evolutionary history. To investigate this hypothesis further, similar study should be run to evaluate decision under risk in children from different cultures. If loss aversion appears to be an ancestral trait, it probably means that some time in our evolution, it improved the quality of decision-making by enhancing survival. In our present world where economics rule many exchanges between individuals, loss aversion sometimes conducts to negative profitability [44]; by case, it may induce investors to keep their investment for too long instead of selling assets [47]; it may also lead to excessive risk-taking, resulting in strong losses [48]–[51]. In this particular context loss aversion may have become a non-adaptive pattern rather than a survival asset.
